# Dual-Target Therapeutic Strategies in Triple-Negative Breast Cancer: Mechanistic Insights and Clinical Potential

**DOI:** 10.3390/cancers17213455

**Published:** 2025-10-28

**Authors:** Meng Yu, Xiaodong Lu

**Affiliations:** 1 School of Pharmaceutical Science and Technology, Hangzhou Institute for Advanced Study, University of Chinese Academy of Sciences, Hangzhou 310024, China; yumeng22@mails.ucas.ac.cn; 2 Institute of Urologic Science and Technology, The First Affiliated Hospital, Zhejiang University School of Medicine, Hangzhou 311121, China

**Keywords:** triple-negative breast cancer, dual-target strategies, drug resistance

## Abstract

**Simple Summary:**

Dual-targeting strategies represent a promising therapeutic approach for triple-negative breast cancer (TNBC), designed to overcome the limitations of single-agent therapies. By concurrently inhibiting critical oncogenic pathways, these combinations can produce synergistic anti-tumor effects and circumvent drug resistance. This review summarizes the challenges of current TNBC regimens and explores the recent advances and clinical prospects of dual-target inhibition.

**Abstract:**

Triple-negative breast cancer (TNBC) is an aggressive malignancy marked by high heterogeneity, metastatic potential, and a lack of established targeted therapies. This review explores emerging dual-target strategies that concurrently address key biological mechanisms in TNBC, including DNA damage repair, cell cycle regulation, epigenetic modulation, metabolic reprogramming, and immune microenvironment remodeling. By acting on multiple signaling nodes, these strategies demonstrate enhanced efficacy, an ability to overcome resistance, and a potential to reverse immunosuppression. Although challenges in patient selection, toxicity, and mechanistic understanding remain—with most strategies in preclinical or early clinical development—this approach offers a promising path toward precision medicine and improved outcomes for TNBC patients.

## 1. Introduction

IARC’s 2022 GLOBOCAN report identifies breast cancer as the leading global cancer, accounting for 11.6% of all cases (2.3 million new diagnoses) and causing approximately 670,000 deaths yearly [1]. Among cancers in female, breast cancer demonstrates the highest incidence and mortality rates and its incidence continues to rise steadily [2]. Breast cancer is a highly heterogeneous malignant tumor originating from breast tissue, characterized by uncontrolled proliferation of breast epithelial cells under the influence of various carcinogenic factors [3]. Based on molecular and pathological features, breast cancer is primarily classified into four major subtypes: Luminal A, Luminal B, human epidermal growth factor receptor 2 (HER2)-enriched, and triple-negative breast cancer (TNBC) [4]. Notably, TNBC, defined by its absence of ER, PR, and HER2 expression, has garnered significant attention due to its distinct molecular profile. It represents 15–20% of all breast cancer cases [5]. This subtype is characterized by strong heterogeneity, high invasiveness, and a high propensity for recurrence and metastasis, leading to elevated mortality rates and the poorest prognosis among all breast cancer subtypes [6]. Based on its molecular heterogeneity, TNBC is further subdivided into six distinct subgroups: basal-like 1 (BL1), basal-like 2 (BL2), mesenchymal (M), mesenchymal stem-like (MSL), immunomodulatory (IM), and luminal androgen receptor (LAR) [7]. While TNBC overall carries the poorest prognosis of all breast cancer types, its molecular subtypes indeed demonstrate distinct clinical outcomes. For instance, emerging evidence suggests the immunomodulatory (IM) subtype is associated with a more favorable prognosis, likely due to higher levels of tumor-infiltrating lymphocytes, whereas the mesenchymal (M) subtype often exhibits more aggressive behavior and poorer outcomes [8,9,10].

Due to the absence of ER and HER2, TNBC is resistant to endocrine therapy and HER2-targeted treatments. Currently, chemotherapy as main treatment for TNBC [11,12], but patients frequently develop drug resistance and result in poor clinical outcomes [13]. In recent years, targeted therapies (such as PARP inhibitors, antibody-drug conjugates) and immune checkpoint inhibitors (such as PD-1/PD-L1 inhibitors) have shown clinical benefits in treating TNBC [14,15]. However, the objective response rates of these monotherapies remain limited, largely due to tumor heterogeneity and activation of compensatory pathways. To address these challenges, dual-target inhibition strategies have gained attention as a promising therapeutic approach for TNBC [16]. By concurrently blocking two oncogenic pathways or simultaneously targeting tumor-intrinsic signaling and the tumor microenvironment, these strategies can more effectively suppress compensatory mechanisms, enhance antitumor efficacy, and mitigate drug resistance [17]. In this review, we systematically summarize recent advances in dual-target inhibition for TNBC, characterizing them into four mechanistic categories: targeting DNA damage repair, cell cycle regulation, epigenetic modulation, and tumor immune microenvironment (TME) remodeling. This comprehensive overview aims to provide a theoretical basis and strategic insights for optimizing precision medicine in TNBC treatment.

## 2. Limitations of Single-Agent Therapies for TNBC

### 2.1. Chemotherapy

Given the unique molecular characteristics of TNBC, chemotherapy remains the cornerstone of treatment, with commonly used agents including anthracyclines, platinum compounds, and taxanes [11]. Chemotherapy is a therapeutic approach that employs chemical agents to eliminate or suppress rapidly dividing cancer cells. Compared with Luminal A or Luminal B subtypes, TNBC patients demonstrate a higher pathological complete response (pCR) rate to neoadjuvant chemotherapy [18]. Compared with chemotherapy, neoadjuvant chemotherapy—administered before surgery—reduces tumor burden, improves surgical outcomes, and lowers the risk of distant metastasis [12]. Standard regimens include AC-T (doxorubicin plus cyclophosphamide followed by paclitaxel), TAC (docetaxel, doxorubicin, and cyclophosphamide), and AC-P (doxorubicin, cyclophosphamide, and carboplatin). Several clinical trials, such as the exploratory FinXX randomized trial [19], GeparTrio trial [20], NeoALTTO trial [21], and CREATE-X trial [22], have shown that neoadjuvant chemotherapy significantly prolongs disease-free survival in TNBC, reinforcing its clinical utility. Moreover, the KYENOTE-522 trial has also demonstrated that adding pembrolizumab to neoadjuvant chemotherapy significantly improved the pCR rate and event-free survival (EFS) compared to chemotherapy alone in early-stage TNBC [23]. However, these benefits are largely confined to non-metastatic patients, and overall survival rates remain unsatisfactory. TNBC is still associated with high risks of recurrence, early metastasis, and poor prognosis. Moreover, prolonged chemotherapy often reduces patient compliance and tolerance, while declining drug sensitivity can limit therapeutic efficacy and foster resistance [13]. The emergence of drug resistance not only predisposes to relapse but may also facilitate metastatic dissemination to vital organs, ultimately resulting in organ failure and mortality [24].

### 2.2. Targeted Therapy: PARP Inhibitors and Antibody–Drug Conjugates

With increasing insights into the molecular biology of TNBC, precision targeted therapies based on molecular subtyping are emerging as important treatment strategies. Garrido et al. analyzed TCGA datasets and genomic libraries from post-neoadjuvant chemotherapy patients and found that over 90% harbored alterations in specific signaling pathways, highlighting the potential for population-specific therapeutic approaches [25]. Several of these dysregulated pathways are targetable with either approved drugs or agents in clinical development, including cell cycle inhibitors, DNA repair enzyme inhibitors, anti-angiogenic agents, PI3K/AKT/mTOR modulators, MAPK/ERK pathway inhibitors, and antibody–drug conjugates (ADCs) [26]. The Poly (ADP-ribose) polymerase (PARP) inhibitor olaparib was the first targeted therapy approved by the FDA for TNBC patients with BRCA1/2 mutations [27]. By inhibiting PARP, a key enzyme in DNA repair, olaparib induces a “synthetic lethal” effect, promoting genomic instability and apoptosis in tumor cells [11]. However, its clinical utility is restricted to patients with BRCA mutations, and long-term treatment often leads to acquired resistance [14]. ADCs represent another promising avenue for advanced TNBC [28]. These agents link monoclonal antibodies to cytotoxic payloads, enabling selective delivery of chemotherapy to tumor cells while limiting off-target toxicity [29]. Sacituzumab govitecan, the first Trop-2–directed ADC, conjugates SN-38 (a topoisomerase I inhibitor) to an anti–Trop-2 antibody, resulting in DNA damage and apoptosis. In the phase III ASCENT trial, sacituzumab govitecan significantly improved both progression-free and overall survival in advanced TNBC, demonstrating meaningful clinical benefit [30]. Nonetheless, TNBC heterogeneity leads to variable antigen expression, potentially reducing target efficiency. Moreover, the tumor microenvironment and treatment-related toxicities, including hepatotoxicity and hematologic adverse effects, further limit the broad clinical application of ADCs.

### 2.3. Immunotherapy

As a novel therapeutic strategy for TNBC, immunotherapy has shown significant clinical potential in recent years. Among immunotherapeutic agents, immune checkpoint inhibitors (ICIs) have achieved the greatest success. These drugs function by blocking inhibitory receptors such as cytotoxic T-lymphocyte antigen 4 (CTLA-4) and programmed cell death protein 1 (PD-1), thereby enhancing the proliferation and cytotoxicity of tumor-infiltrating lymphocytes (TILs) and restoring antitumor immune activity [31,32]. Clinically approved ICIs include anti–PD-1 antibodies (pembrolizumab, nivolumab), anti–PD-L1 antibodies (atezolizumab), and anti–CTLA-4 antibodies (ipilimumab), all of which have demonstrated durable responses in various cancers [33]. In TNBC, early clinical evidence also supports the activity of ICIs. For instance, the phase Ib KEYNOTE-012 trial reported that pembrolizumab monotherapy achieved an overall response rate (ORR) of 18.5% in PD-L1–positive metastatic TNBC, including one complete and four partial responses. The median overall survival was 11.2 months, and 25.9% of patients achieved stable disease, indicating antitumor activity in this population [34]. Despite these encouraging findings, immunotherapy faces significant limitations. Not all patients respond, and both primary and acquired resistance reduce long-term efficacy. The mechanisms driving resistance remain incompletely understood, but the therapeutic outcome is highly dependent on host immune status and the tumor immune microenvironment, leading to substantial interpatient variability in response. Moreover, the high cost of immunotherapy imposes additional barriers to its widespread clinical application.

In summary, although novel therapeutic strategies for TNBC continue to emerge, their clinical progression remains restricted by efficacy, drug resistance, adverse reactions, and economic burden (Table 1). These challenges highlight the urgent need for more effective approaches. In recent years, combination strategies—particularly multi-drug and multi-target regimens—have shown distinct advantages, including synergistic antitumor effects, delayed or reversed resistance, and modulation of the tumor microenvironment. Dual-target inhibition, with its capacity to simultaneously disrupt multiple oncogenic pathways and immune evasion mechanisms, is increasingly recognized as a promising direction to improve TNBC treatment outcomes.

This table summarizes the mechanisms, characteristics, and common adverse reactions of the primary treatment options for Triple-Negative Breast Cancer (TNBC). While chemotherapy remains the current cornerstone of treatment, the optimal therapeutic strategy must be tailored to the individual, based on molecular subtyping, biomarker status, and patient-specific factors.

## 3. Dual-Target Therapeutic Strategies for TNBC

### 3.1. Overcoming PARP Inhibitor Resistance via DNA Damage Repair Co-Targeting

TNBC is characterized by profound genomic instability, frequently associated with defects in homologous recombination repair (HRR). This intrinsic vulnerability renders the DNA damage response (DDR) pathway a critical therapeutic target in TNBC. While DDR dysfunction accelerates genomic instability and drives tumor progression, TNBC cells remain dependent on DDR mechanisms to tolerate replication stress and DNA damage caused by chemotherapy or radiotherapy [35,36]. Thus, therapeutic inhibition of DDR can impair DNA repair capacity and sensitize tumor cells to treatment.

PARP, a key enzyme in single-strand break (SSB) repair, has been clinically targeted with PARP inhibitors for TNBC patients harboring BRCA1/2 mutations [27]. However, not all TNBC patients carry BRCA mutations, and resistance to PARP inhibitors frequently emerges, prompting the development of dual-target strategies to expand their applicability and overcome resistance. For example, Julie L. Boerner et al. demonstrated that combining a topoisomerase I inhibitor—which induces SSBs and replication fork stalling—with a PARP inhibitor blocks base excision repair, leading to SSB accumulation, conversion into double-strand breaks (DSBs), and apoptosis, thereby enhancing antitumor efficacy in TNBC cells [37].

Dual inhibition of PARP and ataxia telangiectasia and Rad3-related protein (ATR) has also shown synergistic effects. ATR is essential for genomic stability and checkpoint control. The dual-target inhibitor PAB-13 suppresses PARP1-mediated SSB repair while attenuating ATR-driven DDR and G_2_/M checkpoint function, resulting in uncontrolled SSB accumulation, conversion to DSBs, aberrant mitosis, and ultimately mitotic catastrophe and apoptosis [38,39].

Another promising approach is the dual inhibition of PARP and nicotinamide phosphoribosyltransferase (NAMPT), the rate-limiting enzyme in NAD^+^ biosynthesis. Since NAD^+^ is required for PARP catalytic activity, NAMPT inhibition not only impairs PARP function but also reduces expression of HRR proteins such as BRCA1 and Rad51. By downregulating BRCA1, NAMPT inhibition artificially induces a homologous recombination deficiency (HRD) state, often termed “BRCAness,” in BRCA1-wild-type tumors. This synthetic HRD phenotype sensitizes these otherwise PARPi-resistant cells to PARP inhibitors, mimicking the synthetic lethality observed in BRCA-mutant TNBC. Mao et al. reported that this strategy synergistically induces DNA damage, apoptosis, and inhibition of TNBC cell migration, significantly suppressing tumor proliferation and metastasis [40].

In recent years, DNA polymerase theta (Polθ) has emerged as a promising therapeutic target in TNBC. Research by Diana Zatreanu et al. demonstrated that the allosteric Polθ inhibitor ART558 induces synthetic lethality in BRCA-deficient TNBC models and overcomes resistance to PARP inhibitors. The inhibitor specifically recognizes the “closed conformation” adopted by Polθ when bound to a DNA/DNA primer–template substrate, and through an induced-fit mechanism, traps the enzyme on DNA for a prolonged duration, thereby disrupting its DNA repair function. This unique “enzyme–DNA trapping” mechanism acts synergistically with PARP inhibition, significantly enhancing synthetic lethality in homologous recombination-deficient tumors. This work not only validates the therapeutic targeting of DNA repair-associated polymerases, but also provides new directions for clinical strategies and small-molecule drug development in TNBC [41,42].

Collectively, these dual-target strategies disrupt multiple key nodes of the DDR pathway, overcoming the traditional dependence of PARP inhibitors on BRCA-mutant tumors and providing new avenues to overcome resistance. Furthermore, combinations of PARP inhibitors with inhibitors of STAT3, RAS, and other signaling pathways have also demonstrated synergistic effects. While these combinations do not directly inhibit DDR, they disrupt complementary processes such as survival signaling, cell cycle progression, and apoptosis evasion, thereby indirectly enhancing PARP inhibitor efficacy and overcoming resistance [43,44].

### 3.2. Targeting CDKs and Cooperative Pathways for TNBC Therapy

Under normal physiological conditions, cell cycle progression is tightly regulated by cyclin-dependent kinases (CDKs) and their associated cyclins. In TNBC, however, the G_1_–S transition is markedly accelerated, accompanied by aberrant expression of multiple CDK family members, including CDK1, CDK2, CDK4, and CDK6 [45,46]. Functional studies have shown that inhibition of CDK1/2 induces cell cycle arrest and apoptosis, while selective inhibition of CDK4/6 primarily triggers G_1_-phase arrest [47,48]. Despite the clinical use of CDK4/6 inhibitors, resistance frequently emerges in TNBC. Consequently, current research has shifted toward dual-target strategies that either co-inhibit multiple CDKs or combine CDK inhibition with other signaling pathways to enhance efficacy and overcome resistance [49]. The representative strategies currently reported in research include: (1) Targeting the DNA damage response (DDR) by combinations of CDK12/PARP and CDK6/PARP inhibitors to co-induce synthetic lethality; (2) Targeting epigenetic regulators, such as CDK9/EZH2 and CDK7/HDAC1 inhibitors, which synergistically disrupt oncogenic transcription and gene silencing; and (3) Targeting signaling transduction pathways, for example, with CDK4/6/PI3Kα inhibitors that concurrently block mitogenic signaling and cell cycle progression.

One such approach was described by Wang et al., who demonstrated that in TNBC models treated with the CDK4/6 inhibitor Palbociclib, dual inhibition of STAT3 and CDK2 produced synergistic antitumor effects. The dual STAT3/CDK2 inhibitor Nifuroxazide suppressed STAT3 phosphorylation, thereby blocking the release of Palbociclib-induced senescence-associated secretory phenotype (SASP) and alleviating SASP-mediated remodeling of the tumor microenvironment, metastasis, and drug resistance. At the same time, Nifuroxazide directly targeted CDK2, disrupting its interaction with Cyclin E1 and promoting autophagic degradation of CDK2. Together, these effects synergized with Palbociclib to induce G_1_ arrest, senescence, and potent inhibition of tumor proliferation and metastasis, overcoming limitations of Palbociclib monotherapy [50].

Simultaneous inhibition of CDK2 and CDK4 has also shown synergistic potential. The natural small-molecule compound 4-AAQB directly binds to CDK2 and CDK4, inhibits their kinase activities, and downregulates cyclins D, E, and A, leading to G_1_/S arrest. Importantly, 4-AAQB also suppresses the DDR pathway by impairing activation of key repair proteins including BRCA1, pCHEK1, and pCHEK2, which results in γH2AX accumulation and apoptosis. This strategy simultaneously blocks cell cycle progression and DNA damage repair, thereby overcoming compensatory repair mechanisms and resistance to conventional therapies [51].

In addition to CDKs, bromodomain-containing protein 4 (BRD4), a member of the BET family, is a key transcriptional and epigenetic regulator of genes involved in G_1_/S transition. Dual inhibition of BRD4 and CDK4/6 induces mitotic errors, genome doubling, and aneuploidy, ultimately driving G_1_ arrest and a senescence-like phenotype. This combination not only enhances the antitumor activity of CDK4/6 inhibitors but also overcomes intrinsic resistance mechanisms through transcriptional reprogramming. The resistance mechanism is characterized by upregulation of pro-proliferative genes (e.g., CCND1, CCNE1) and downregulation of cell cycle inhibitors (e.g., CDKN1A, RB1). Such dysregulation of gene expression is considered a key mechanism by which tumor cells evade cell cycle arrest and develop resistance [49,52].

Finally, CDK inhibitors can also be combined with non–cell cycle targets to broaden their therapeutic potential. For instance, dual inhibition of CDK4/6 and CDK7 suppresses the FOXM1–SREBP1–p300 axis, thereby repressing transcriptional activation and histone modification of key cholesterogenic genes. This cross-pathway approach synergistically inhibits TNBC proliferation and overcomes resistance [53]. Collectively, these findings underscore the versatility of CDK inhibitors and the promise of dual-target therapies that exploit cell cycle vulnerabilities. By disrupting compensatory CDK activity, integrating DNA damage repair inhibition, or engaging parallel oncogenic pathways, such approaches significantly expand the therapeutic scope of CDK inhibition and offer novel avenues for precision TNBC treatment.

### 3.3. Dual-Targeting Epigenetic Regulators: DNMT, HDAC, and Beyond

Epigenetic dysregulation is a fundamental driver of tumorigenesis and cancer progression, modulating gene expression without altering the DNA sequence. This regulation encompasses DNA methylation, diverse histone modifications (such as acetylation, methylation, and ubiquitination), and non-coding RNA–mediated control. In TNBC, aberrant epigenetic modifications—including abnormal DNA methylation, imbalanced histone modifications, and dysfunction of regulatory factors such as BET proteins—promote disease progression and confer treatment resistance. For instance, hypermethylation of tumor suppressor gene promoters, along with dysregulated histone methyltransferases and histone deacetylases (HDACs), impairs tumor cell survival control and enhances malignant phenotypes. Accordingly, targeting epigenetic modifications and their downstream pathways has emerged as a promising therapeutic strategy in TNBC [54].

Two key classes of epigenetic enzymes, DNA methyltransferases (DNMTs) and HDACs, play central roles in shaping the TNBC epigenetic landscape. DNMTs catalyze DNA methylation, leading to silencing of tumor suppressor genes, whereas HDACs remove acetyl groups from histones and non-histone proteins, compact chromatin, and modulate transcription factor activity [55,56,57,58]. Given their cooperative contributions to tumorigenesis, epithelial–mesenchymal transition (EMT), and immune microenvironment modulation, dual inhibition of DNMT and HDAC has gained attention as a strategy to overcome the limitations of monotherapies.

For example, the dual-target inhibitor J208 concurrently inhibits DNMT and HDAC in TNBC, leading to transcriptional activation of endogenous retroviruses (ERVs) and accumulation of double-stranded RNA (dsRNA). This activates the RIG–I–MAVS pathway, induces type I/III interferon expression, and triggers innate immune responses via the JAK–STAT pathway. Consequently, J208 induces G_1_-phase arrest and apoptosis, suppresses tumor proliferation and migration, and enhances tumor immunogenicity through a “viral mimicry” effect, achieving dual antitumor activity via epigenetic reprogramming and immune activation [59]. Dual DNMT/HDAC inhibition also reverses the EMT phenotype. By inhibiting EpCAM and Wnt signaling, it downregulates mutant p53, ZEB1, and EZH2, while upregulating E-cadherin and H3K27me3. This suppresses tumor cell proliferation, migration, invasion, and stemness, and induces apoptosis, overcoming the limitations of HDAC monotherapy in controlling metastasis and mitigating potential pro-invasive risks [60].

Beyond DNMT/HDAC co-targeting, other dual-target strategies show considerable promise. For instance, simultaneous inhibition of topoisomerase II and HDAC modulates the immune microenvironment, enhances infiltration of lymphocytes and B cells, and reinstates antitumor immune responses, addressing the constraints of HDAC monotherapy in tumor growth suppression and resistance reversal [16,61]. Additional combinations, such as HDAC/G-quadruplex, HDAC/CDC25 phosphatase, HDAC/ROCK, and HDAC/EGFR, synergistically target both epigenetic regulation and cell cycle or downstream signaling pathways, further enhancing antitumor efficacy and offering novel avenues to overcome HDAC inhibitor resistance and therapeutic limitations [62,63,64,65].

### 3.4. Synergistic Targeting of PI3K/AKT/mTOR and MAPK Pathways in TNBC

Alterations in tumor cell metabolic signaling pathways and associated metabolic enzymes have emerged as a key focus in cancer research due to their critical roles in regulating cell proliferation, metastasis, drug resistance, and immune suppression. Metabolic reprogramming, a core mechanism enabling tumor cells to sustain rapid proliferation, is closely linked to tumor progression, invasion, metastasis, and poor clinical prognosis [66]. In TNBC, this reprogramming is characterized by heightened dependence on specific metabolic pathways, including enhanced glycolysis, dysregulated lipid metabolism, and hyperactive glutamine metabolism [67]. Although inhibitors targeting individual metabolic pathways have been developed, monotherapy often triggers compensatory mechanisms, resulting in drug resistance. Consequently, combination strategies targeting multiple metabolic nodes are increasingly considered more effective.

The PI3K/AKT/mTOR signaling pathway is central to TNBC survival and proliferation, coordinating cell cycle progression, inhibiting apoptosis, promoting angiogenesis, and driving metabolic reprogramming. Its activation is associated with poor prognosis, making it a key therapeutic target [68]. While PI3Kα inhibitors (e.g., BYL719) and mTOR inhibitors (e.g., everolimus) show preclinical efficacy, resistance limits monotherapy effectiveness. Cretella et al. demonstrated that combining PI3K/AKT/mTOR inhibitors with the CDK4/6 inhibitor palbociclib synergistically suppresses TNBC growth. This combination simultaneously blocks the PI3K/AKT/mTOR and CDK4/6/Rb/Myc axes, downregulates c-Myc, induces G_1_ phase arrest, triggers apoptosis, and inhibits glucose metabolism under hypoxia, overcoming the limitations of palbociclib monotherapy and providing a novel approach for Rb-positive TNBC patients [69]. Currently, combination therapies targeting the PI3K/AKT/mTOR signaling pathway are typically tailored based on tumor genomic characteristics. For example, in patients with PIK3CA-mutated, HR+/HER2- breast cancer, the combination of a PI3Kα inhibitor with endocrine therapy has demonstrated significant clinical efficacy. The SOLAR-1 trial showed that alpelisib plus fulvestrant nearly doubled the median progression-free survival (PFS) compared to endocrine therapy alone (11.0 months vs. 5.7 months), establishing this regimen as a standard treatment option after progression on CDK4/6 inhibitors. Furthermore, the BYLieve study confirmed that switching to a PI3Kα inhibitor combined with endocrine therapy following CDK4/6 inhibitor treatment continues to provide substantial antitumor activity. over 50% of patients with PIK3CA mutations remained progression-free at 6 months after switching to alpelisib plus fulvestrant upon CDK4/6 inhibitor failure.

Mitogen-Activated Protein Kinases (MAPKs) also play pivotal roles in TNBC signaling. The p38 MAPK pathway, activated by cellular stress stimuli such as UV radiation, heat shock, osmotic stress, and cytokines, regulates gene expression and cellular functions [70]. Dual targeting to inhibit AKT phosphorylation while activating p38 MAPK can block autophagosome-lysosome fusion, enhancing paclitaxel chemosensitivity, suppressing tumor proliferation, metastasis, and stemness, and mitigating toxicity associated with traditional autophagy inhibitors [71]. Similarly, the MEK/ERK pathway, a major branch of MAPK signaling involving RAS, RAF, MEK, and ERK, regulates proliferation, differentiation, migration, apoptosis, and gene expression [72]. Concurrent inhibition of MEK5/ERK5 and PI3K/AKT in TNBC synergistically blocks survival signals, leading to dephosphorylation of the pro-apoptotic protein Bad (Ser112 and Ser136), Caspase-3 activation, and apoptosis, thereby overcoming the limitations of single-pathway inhibition due to compensatory crosstalk [73]. Additionally, dual targeting of upstream and downstream nodes within the RAS-MAPK pathway, such as RAF and MEK, effectively promotes apoptosis, inhibits proliferation, and circumvents monotherapy resistance and feedback activation [74]. In summary, dual-target strategies that concurrently modulate tumor metabolism–related signaling pathways represent a promising avenue for improving therapeutic efficacy in TNBC.

## 4. Other Potential Dual-Target Inhibition Strategies

While dual-target strategies against classical pathways such as DDR, cell cycle regulation, epigenetic modification, PI3K/AKT/mTOR, MAPK, and PARP have shown potential in TNBC treatment, most remain in early-stage investigation. Given the high heterogeneity and complex drug resistance mechanisms of TNBC, there is an urgent need to develop more diversified combination therapies.

Beyond the strategies discussed above, combining ICIs with other targeted therapies has emerged as a promising approach. Anti-PD-1/PD-L1 therapy, one of the most clinically advanced ICIs, has shown limited response rates in TNBC due to primary resistance, insufficient T-cell infiltration, and the immunosuppressive “cold tumor” microenvironment. The transforming growth factor-beta (TGF-β) signaling pathway plays a pivotal role in modulating ICI efficacy [75,76,77,78]. TGF-β activates cancer-associated fibroblasts (CAFs), promotes excessive collagen deposition, and forms a dense stromal barrier that restricts T-cell infiltration. It also suppresses cytotoxic T cell and NK cell activity while recruiting regulatory T cells (Tregs), collectively promoting immune evasion. Yi et al. demonstrated that a bispecific antibody targeting both TGF-β and PD-L1 exerts synergistic antitumor effects by concurrently blocking TGF-β-Smad and PD-1/PD-L1-NFAT signaling. This bispecific antibody inhibits CAF activity, reduces extracellular matrix barriers, enhances CD8^+^ T-cell infiltration, promotes T cell and NK cell activation, and improves dendritic cell maturation and antigen presentation, effectively converting “immune-excluded” tumors into “immune-inflamed” ones [79]. Similarly, a bispecific antibody targeting Siglec-15 (S15) and TGF-β leverages tumor-specific S15 expression to localize TGF-β inhibition, synergistically overcoming immunosuppression and remodeling the tumor immune microenvironment [80]. These approaches address the limitations of single-target therapies caused by insufficient targeting or redundant immunosuppressive mechanisms. In addition, combinations of anti-PD-1/PD-L1 antibodies with chemotherapy, CDK inhibitors, or type I interferon inducers have demonstrated promising synergistic antitumor effects [81,82,83].

Dual-target strategies also extend to protein kinase modulation. For example, combining the PKC inhibitor enzastaurin with paclitaxel overcomes paclitaxel resistance in TNBC by activating the GCN2–p-eIF2α signaling axis, promoting Aurora kinase B accumulation, restoring mitotic arrest, and inducing apoptosis [84]. Likewise, simultaneous inhibition of threonine–tyrosine kinase (TTK/MPS1) and polo-like kinase 1 (PLK1) disrupts spindle assembly checkpoint integrity, causing aberrant mitotic exit, chromosome segregation errors, aneuploidy, and potent antiproliferative effects [85].

In addition, peptide-drug conjugates (PDCs) is also an advanced form of targeted that aligns with the core principles of dual-targeting: enhancing efficacy and reducing toxicity through coordinated multi-component action. Research has demonstrated that conjugating docetaxel with the TH19P01 peptide creates a peptide-drug conjugate (TH1902) for targeted cancer therapy. Although inactive alone, TH19P01 is efficiently internalized into TNBC cells via the SORT1 receptor. This delivers docetaxel precisely into tumors, where it strongly inhibits proliferation and migration, disrupts microtubule dynamics, and downregulates the anti-apoptotic protein Bcl-xL to promote cell death. Compared to free docetaxel, TH1902 offers greater efficacy and a superior safety profile without causing significant neutropenia [86]. While mechanistically distinct from combining two free drugs, PDCs such as TH1902 fundamentally represent a dual-entity system where the peptide provides precise targeting (engaging SORT1) and the cytotoxic payload (docetaxel) provides the therapeutic effect, creating a powerful synergistic unit within a single molecule.

Although various dual-target strategies for TNBC have been explored, the majority are still in the preclinical phase. These investigations typically establish a rationale for target pairing and subsequently substantiate the feasibility and synergistic therapeutic potential of the combinations through a series of in vitro and in vivo studies.

However, several combination regimens involving immunotherapy, chemotherapy, AKT inhibitors, and ADCs have advanced into Phase II or III clinical trials, demonstrating a clear trajectory toward clinical application. For instance, in the neoadjuvant setting, the KEYNOTE-522 trial (pembrolizumab plus paclitaxel/carboplatin; Merck) and the IMpassion031 trial (atezolizumab plus nab-paclitaxel; Roche) have both demonstrated significant improvements in pathological complete response (pCR) and event-free survival (EFS), establishing immune–chemotherapy as a standard of care for early-stage high-risk TNBC [23,87]. Furthermore, the ASCENT-03/04 trial series (Gilead Sciences), which evaluates sacituzumab govitecan in combination with pembrolizumab for advanced TNBC, has shown preliminary data indicating superior Objective Response Rate (ORR) and progression-free survival (PFS) compared with historical controls [88]. In the realm of novel therapeutic modalities, the PD-L1/VEGF bispecific antibody BNT327 (PM8002; BioNTech) has completed early-phase dose escalation and entered randomized Phase III evaluation, attracting attention for its potential synergistic mechanism [89]. In addition, a Phase Ib/II clinical study (Poster #398P) is evaluating the FAK inhibitor IN10018 in combination with pegylated liposomal doxorubicin (PLD) and the anti-PD-1 antibody toripalimab for the treatment of advanced TNBC. Available clinical data indicate that this triple-combination regimen demonstrates significant synergistic antitumor activity and survival benefits, along with a favorable safety and tolerability profile. These findings suggest that, beyond dual-target inhibitor strategies, multi-target combination therapies represent a promising direction for future therapeutic development in TNBC.

Despite these promising outcomes, the safety profiles of such combination therapies warrant careful investigations. The potential treatment-related adverse events—including diarrhea, hepatotoxicity (e.g., elevated transaminases), cutaneous rash, peripheral neuropathy, and myelosuppression—need to be paid attention, which may require dose adjustment or treatment discontinuation. Therefore, the development of risk prediction models and individualized management strategies will be essential to optimize the therapeutic balance between efficacy and safety. The key dual-target strategies in TNBC are shown in Table 2.

This table systematically summarizes dual-target therapeutic strategies for TNBC, spanning key areas such as DNA damage repair, the immune microenvironment, and metabolic signaling. While most strategies are preclinical, they aim to overcome drug resistance, expand patient eligibility, and enhance anti-tumor efficacy, thereby advancing precision oncology for TNBC.

**Figure 1:**
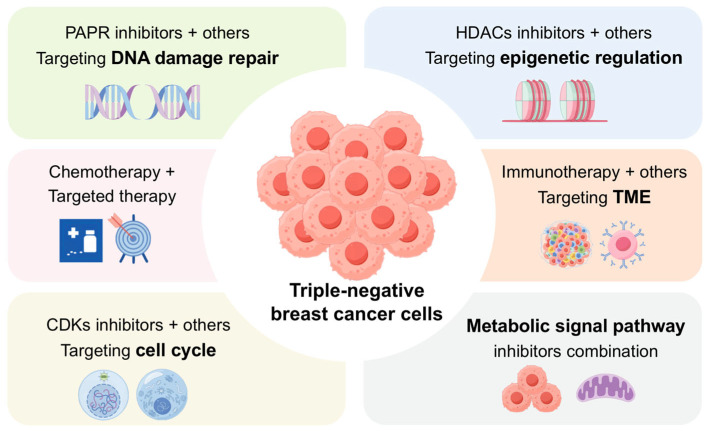
Schematic of Dual-Target Therapeutic Strategies in TNBC. The schematic overview depicts rational combinations of chemotherapeutic, targeted, and immunotherapeutic agents. The central aim is to achieve synergistic efficacy through the concurrent modulation of non-redundant tumor-promoting pathways.

In summary, dual- and multi-target combination strategies represent an emerging frontier in TNBC therapy. By simultaneously disrupting multiple signaling pathways and cellular processes, these approaches can overcome monotherapy resistance and enhance antitumor efficacy (Figure 1). Continued identification of synergistic target pairs and mechanistic studies will further expand precision therapeutic options for TNBC patients.

## 5. Conclusions and Perspectives

TNBC has poorest prognosis among breast cancer subtypes, posing significant clinical challenges due to its high heterogeneity, aggressive metastatic potential, and lack of well-defined therapeutic targets. Current clinical management relies predominantly on chemotherapy. While temporarily effective, this approach is often associated with drug resistance and adverse effects such as myelosuppression, cardiotoxicity, and neurotoxicity, limiting both efficacy and patient quality of life. These limitations underscore the urgent need for novel therapeutic strategies.

In recent years, multi-drug and multi-target strategies have shown significant promise in TNBC treatment, complementing the development of single-target agents. By simultaneously enhancing therapeutic efficacy, overcoming resistance, and modulating the tumor microenvironment, these approaches, particularly dual-target strategies, have become a major research focus.

This review systematically summarizes dual-target strategies against critical biological processes in TNBC, including DNA damage repair, cell cycle regulation, epigenetic modification, tumor metabolic signaling, and immune microenvironment remodeling. These strategies aim to overcome the limitations of monotherapy by synergistically modulating multiple signaling pathways and cellular mechanisms. For example, combined targeting of CDK4/6 with STAT3 or CDK2, concurrent inhibition of BRD4 and CDK4/6, and dual immune checkpoint blockade with TGF-β inhibition have all demonstrated significant synergistic antitumor effects in preclinical models.

Despite these encouraging findings, the clinical translation of dual-target strategies faces several challenges. First, a deeper, functional understanding of TNBC heterogeneity is paramount. Research must evolve from static molecular subtyping to dynamically mapping the crosstalk between genomic drivers, epigenetic states, and the tumor immune microenvironment under therapeutic pressure. This will uncover context-specific vulnerabilities and guide the shift from empirical combinations to mechanism-driven target selection. Second, the discovery and validation of predictive biomarkers is a non-negotiable prerequisite for success. Although it has been established that some important biomarkers like TROP2 (detectable via liquid biopsy) and Tumor-Infiltrating Lymphocytes (TILs), future efforts should focus on developing composite signatures that integrate DNA repair deficiencies (e.g., BRCAness), epigenetic markers, immune cell profiles, and liquid biopsy data to enable ultra-precise patient stratification and overcome resistance. Third, technological innovation will be a major catalyst. This includes the rational design of novel modalities like bispecific antibodies, bifunctional degraders (PROTACs), and peptide-drug conjugates; the development of smart nanocarriers for co-delivery of synergistic agents; and the systematic use of advanced preclinical models like patient-derived organoids (PDOs) and xenografts (PDXs) to improve the predictive power of drug synergy.

Nonetheless, the continued development of dual-target strategies represents a promising direction for precision medicine in TNBC. Current clinical practice already demonstrates the value of combining chemotherapy with targeted therapies or immunotherapy to improve efficacy and reduce toxicity, supporting the feasibility of dual-target approaches. Supported by the discovery of novel targets, innovations in drug delivery, and optimized preclinical models like organoids and patient-derived xenografts, the translational application of dual-targeting strategies is poised to advance significantly. In summary, with further mechanistic insights and clinical validation, these approaches hold considerable potential to improve outcomes and offer new hope for patients with this aggressive, treatment-refractory disease.

## Figures and Tables

**Table 1:** Overview of the key mechanisms, characteristics, and adverse reactions of current TNBC therapies.

Treatment Category	Key Agents	Mechanism	Clinical Benefits	Adverse Effects
**Chemotherapy**	Anthracyclines (Doxorubicin, Epirubicin)	Inhibit topoisomerase II and disrupt DNA/RNA synthesis	Broad-spectrum efficacy, established clinical use, high response rates	Cardiotoxicity, myelosuppression, vomiting
	Taxanes (Paclitaxel, Docetaxel)	Stabilize microtubules and arrest mitosis	High efficacy, improves pCR, well-tolerated in patients	Peripheral neuropathy, hypersensitivity reactions, myelosuppression
	Platinum agents (Carboplatin, Cisplatin)	Form DNA adducts, induce DNA damage and apoptosis	BRCA-mutation effective, synergistic with other DNA-damaging agents	Nephrotoxicity, ototoxicity, myelosuppression
	Cyclophosphamide	Interfere with DNA synthesis, inhibit DNA replication, immunosuppression	Versatile, multiple combinations, oral option	Myelosuppression, hemorrhagic cystitis, nausea and vomiting
**Targeted Therapy**	PARP inhibitors (Olaparib, Talazoparib)	Inhibit PARP, impair DNA damage repair, and induce “synthetic lethality”	BRCA-specific, better tolerability, oral administration	Limited to BRCA1/2 mutation carriers; anemia, nausea; drug resistance
	ADC drugs (Sacituzumab Govitecan)	Precisely deliver cytotoxins and directly kill tumor cells	Targeted delivery, improved efficacy, reduced toxicity	Hematological/neurotoxicity and liver damage
	PI3K/AKT/mTOR inhibitors (Everolimus)	Block PI3K/AKT/mTOR signaling, inhibit tumor proliferation and survival	Pathway-specific, biomarker-driven, precision therapy	Hyperglycemia, blood toxicity and gastrointestinal reactions
	Anti-angiogenic agents (Bevacizumab)	Target VEGF, inhibit tumor angiogenesis	Synergistic with chemotherapy, delays disease progression	Hypertension, proteinuria and thrombosis
	EGFR inhibitors (Cetuximab)	Block EGFR signaling, inhibit tumor growth/metastasis	EGFR-targeted, specific approach	Dermatological/liver toxicity, hypomagnesemia
**Immunotherapy**	PD-1 or PD-L1 inhibitors (Pembrolizumab, Atezolizumab)	Block PD-1/PD-L1 axis, reverse T-cell inhibition and restore anti-tumor immune response	Durable responses, long-term control, multiple cancer types	Gastrointestinal reactions, flu-like symptoms and immune related adverse reactions
	CTLA-4 inhibitors (Ipilimumab)	Block CTLA-4 signaling, enhance T-cell activation and proliferation	Potent T-cell activation with anti-PD-1/PD-L1 agents, immune enhancement	Gastrointestinal symptoms, immune related adverse reactions, and endocrine disorders

**Table 2:** Dual-target therapeutic strategies for TNBC.

Dual-Target Strategy	Experimental Models Used	Key Agents	Key Outcomes	Proposed Mechanism
**PARP + Topoisomerase I**	**In vitro:** SUM149, SUM159, SUM1315, HCC1937, MDA-MB-23, MX-1 **In vivo:** MX-1 xenografts	ABT-888 (PARP inhibitor) + CPT-11 (Topoisomerase I inhibitor)	CI values < 1, tumor free on d96 4/6, tumor growth delay 40.5 days, tumor volume decreased	Trigger DNA damage, block damage repair, induce DSBs and result in apoptosis
**PARP + ATR**	**In vitro:** MDA-MB-231, MDA-MB-468, HCC1937, MDA-MB-436, MCF-7 **In vivo:** MD-MB-468 xenografts	PAB-13 (dual-target inhibitor)	Superior Cytotoxicity, γH2AX Increase: >2-fold, tumor growth inhibition ~80% (vs control)	Impair SSB repair and attenuate DDR/G2-M checkpoint, induce mitotic catastrophe
**PARP + NAMPT**	**In vitro:** MDA-MB-231, MDA-MB-436	13j (dual-target inhibitor)	CI values < 1, apoptosis increased, cell migration inhibited	Downregulate NAD^+^/BRCA1/Rad51, promote DSB formation
**PARP + Polθ**	**In vitro:** SUM149, CAL51, COV362, MDA-MB-436 **In vivo:** subcutaneous tumor	Olaparib (PARP inhibitor) + ART558 (Polθ inhibitor)	Enhanced apoptosis/growth inhibition, tumor growth inhibition ~80%	Unique “enzyme–DNA trapping” mechanism
**CDK2 + STAT3**	**In vitro:** MDA-MB-231, 4T1	Nifuroxazide (dual-target inhibitor), Palbociclib (CDK4/6 inhibitor)	CI values < 1, cell proliferation inhibited, cellular senescence induced	Inhibit STAT3-SASP and CDK2-Cyclin E1, induce G1 arrest and senescence
**CDK2 + CDK4**	**In vitro:** MDA-MB-231, Hs578T **In vivo:** orthotopic MDA-MB-231/Hs578T	4-AAQB (dual-target inhibitor)	cell proliferation inhibited, apoptosis increased, tumor volume decreased	Inhibit CDK2/4 and Cyclins D/E/A; suppress DDR, induce γH2AX and apoptosis
**CDK4/6 + BRD4**	**In vitro:** SUM159, SUM149, CAL-51, EMG3, MDA-MB-157, MDA-MB-436 **In vivo:** orthotopic SUM159	Palbociclib (CDK4/6 inhibitor) + JQ1 (BET inhibitor)	Cell cycle arrest, tumor growth inhibited	Induce genomic instability, G1 arrest/senescence, G1-S reprogramming
**HDAC + DNMT**	**In vitro:** BT-549, T-47D, 4T1, HCC1937, MDA-MB-231, MDA-MB-453	J208 (dual-target inhibitor)	Proliferation/migration inhibited, apoptosis increased	Induce ERV/dsRNA and RIG-I–MAVS–IFN pathway activation, modulate EMT markers
**HDAC + Topoisomerase II**	**In vitro:** MDA-MB-231, MDA-MB-468 **In vivo:** subcutaneous tumor	SAHA (HDAC inhibitor) + DOX (Topoisomerase II inhibitor)	CI values < 1, tumor volume/lung metastases decreased	Promote lymphocytes and B cells infiltration, restore antitumor immunity, inhibit tumor growth
**PI3K/AKT/mTOR + CDK4/6**	**In vitro:** MDA-MB-231, MDA-MB-468, HCC-38, MCF-7	PI3K/AKT/mTOR inhibitors + Palbociclib (CDK4/6 inhibitor)	Enhanced growth inhibition, apoptosis increased to 31%,	Downregulate c-Myc, enhance G1 arrest, suppress glucose metabolism
**PI3K/AKT/mTOR + p38 MAPK**	**In vitro:** MDA-MB-231, MCF-7, HBL-100, HCC1937, HCC1954, SK-BR-3 **In vivo:** orthotopic MDA-MB-231	terpenoid tanshinone I + paclitaxel	Colony formation reduced, LC3-II/LC3-I ratio increased 1.72-fold, tumor growth inhibition	Block autophagosome-lysosome fusion
**MEK5/ERK5 + PI3K/AKT**	**In vitro:** MDA-MB-231, BT-549, MDA-MB-468	Ipatasertib (pan-AKT inhibitors) + SC-1–181 (MEK5 inhibitor)	CI values < 1, cell migration inhibited	Block survival signals, induce Bas dephosphorylation and Caspase-3 activation
**TGF-β + PD-L1**	**In vitro:** A549, MCF7, BT474, 4T1 **In vivo:** orthotopic EMT-6-hPDL1 and 4T1- hPDL1	BiTP (a bispecific antibody targeting TGF-β and human PD-L1)	Treg differentiation inhibited, T cell activation enhanced, tumor volume decreased, survival extended	Suppress CAF activity, promote CD8^+^ T-cell/NK/DC infiltration and activation
